# The Synthesis of YNU-5 Zeolite and Its Application to the Catalysis in the Dimethyl Ether-to-Olefin Reaction

**DOI:** 10.3390/ma13092030

**Published:** 2020-04-26

**Authors:** Qing Liu, Yuka Yoshida, Naoto Nakazawa, Satoshi Inagaki, Yoshihiro Kubota

**Affiliations:** Division of Materials Science and Chemical Engineering, Yokohama National University, Yokohama 240-8501, Japan; liu-qing-tf@ynu.jp (Q.L.); yoshidayuka83@gmail.com (Y.Y.); naoto-nakazawa-bd@tosoh.co.jp (N.N.); inagaki-satoshi-zr@ynu.ac.jp (S.I.)

**Keywords:** zeolite, YNU-5, solid acid catalyst, DTO reaction

## Abstract

During prior investigations of the synthesis of the novel zeolite YNU-5 (**YFI**), it was found that a very slight amount of an impurity phase contaminated the desired zeolitic phase. This impurity was very often ZSM-5 (**MFI**). The phase composition was determined to be sensitive to the water in the synthesis mixture, and it was possible to obtain a pure phase and also to intentionally generate a specific impurity phase. In the present work, trials based on the dimethyl ether-to-olefin (DTO) reaction using a fixed-bed downflow reactor were performed to assess the effect of the purity of YNU-5 on its catalytic performance. Dealuminated pure YNU-5 exhibited rapid deactivation due to coking at time on stream (TOS) values exceeding 5 min. Surprisingly, this deactivation was greatly suppressed when the material contained a trace amount of ZSM-5 consisting of nano-sized particles. The formation of ZSM-5 nanoparticles evidently improved the performance of the catalytic system during the DTO reaction. The product distributions obtained from this reaction using highly dealuminated and very pure YNU-5 resembled those generated by 12-ring rather than 8-ring zeolite catalysts. The high selectivity for desirable C3 and C4 olefins during the DTO reaction over YNU-5 is beneficial.

## 1. Introduction

Zeolites are crystalline aluminosilicates that possess an exceptional combination of properties, including high thermal stability, Brønsted acidity, and microporosity, due to their well-defined channel systems [1,2]. These materials have been used in a diverse range of applications, including as ion exchangers, adsorbents, and catalysts for many refining and petrochemical processes [3,4,5,6]. Even though there are a variety of chemical compositions in zeolite-like materials, aluminosilicates are the most typical. Zeolites possessing a high Si/Al ratio and a three-dimensional (3D) channel system including large pore (that is, 12-ring) channels are of particular interest, since they combine high thermal and hydrothermal stability with superior resistance to pore blockage [7,8]. Some examples of high-silica, large-pore microporous aluminosilicates that are especially promising on the basis of the above criteria are beta (*BEA) [8,9,10,11,12,13], MCM-68 (**MSE**), and related **MSE**-type materials. Another interesting example is CIT-1 (**CON**), which has a 12-12-10-ring pore system. This material was first synthesized as a borosilicate [14], after which the Al-containing borosilicate **CON** ([Al,B]-**CON**) was successfully crystallized [15]. Al-containing **CON**-type zeolites have exhibited excellent catalytic performance in the methanol-to-olefin (MTO) reaction because of their unique structures and their ability to promote the ready accessibility and diffusion of reactants [15,16,17].

Some small-pore (that is, 8-ring) zeolite frameworks such as **CHA** (having an 8-8-8-ring system) can also be applied to the MTO reaction, and **CHA** can also be used as a catalyst for the NH_3_-selective catalytic reduction (SCR) reaction. Very recently, our own group has found that the **AFX** framework (having an 8-8-8-ring structure) is an even more interesting catalyst for the NH_3_-SCR process [18]. In addition, it should be noted that mordenite (**MOR**) has 12-ring straight channels with intersecting 8-ring pores, and is one of the most industrially useful zeolite catalysts. It is thus expected that combinations of 12-ring and 8-ring pores will provide useful materials.

Our group has successfully synthesized the new aluminosilicate zeolite YNU-5 having a 12-12-8-ring pore system together with an independent 8-ring system, using dimethyl- dipropylammonium (Me_2_Pr_2_N^+^) as the organic structure-directing agent (OSDA) [19]. This material was given the framework type code **YFI** by the International Zeolite Association (IZA) [20]. After the synthesis and structural determination of this substance, it was characterized with regard to potential catalytic applications in three respects: (1) by obtaining information regarding critical factors that affect the successful crystallization of YNU-5, (2) by establishing framework stabilization when preparing a high-silica YNU-5 catalyst, and (3) by assessing the relationship between phase purity (that is, the presence of very small amounts of an impurity phase) and catalytic performance.

Regarding issue (2), our work demonstrated that YNU-5 only crystallizes at a Si/Al ratio of approximately 9 in the product. Although dealumination was possible using a liquid phase post-synthetic treatment with nitric acid, the framework stability of this material was determined to be sensitive to the dealumination conditions. Specifically, a reflux temperature (oil-bath temperature 130 °C rather than 80 °C) promoted framework stability by allowing the migration of Si to fill site defects generated by the removal of framework Al [21]. This simple technique for stabilizing the framework is very valuable because it guarantees the thermal and hydrothermal stability of the catalyst during use and/or regeneration. A remaining challenge that corresponds to issue (1) is that the synthesis window is very narrow, and in most cases, a very slight amount of an impurity phase contaminates the desired YNU-5 product. In our previous work [21], several competing phases, such as MCM-68 (**MSE**), ZSM-5 (**MFI**), and beta (*BEA), were observed following slight changes in the gel composition (especially the amount of water) during synthesis. Even after optimization of the synthesis conditions, trace amounts of **MFI** or **MOR** were frequently still observed as an impurity.

To address issue (3), we focused on the catalytic performance of YNU-5 with regard to the dimethyl ether (DME)-to-olefin (DTO) reaction. This reaction, together with the MTO reaction, is important as an alternative to the thermal cracking of ethane (supplied from nonpetroleum fossil-based resources such as natural gas or shale gas) because thermal cracking alone cannot satisfy the demand for propylene. In addition, butenes and higher molecular weight olefins are currently more in demand than ethylene. During these acid-catalyzed reactions, we found that even trace amounts of impurities in the YNU-5 catalyst greatly affected the catalytic performance, which was unexpected because this effect is not often observed. Therefore, in order to employ this material in catalytic applications, it is essential to establish how these impurities affect the performance of YNU-5. 

We report here additional systematic investigations of the optimal synthetic conditions (specifically, gel composition, crystallization state, and time span) and of the effect of small concentrations of **MFI** impurities on the catalytic performance of YNU-5 during the DTO reaction.

## 2. Materials and Methods 

### 2.1. Measurements

Powder X-ray diffraction (XRD; Ultima-IV, Rigaku, Akishima, Tokyo, Japan) data were collected using Cu Kα radiation and operating at 40 kV and 20 mA to examine the crystallinity and phase purity of the zeolite catalysts. The Si/Al molar ratios in the bulk materials were determined by inductively coupled plasma—atomic emission spectrometry (ICP-AES; ICPE-9000, Shimadzu Ltd., Kyoto, Japan). In preparation for these analyses, a catalyst sample (20 mg) was suspended in Milli-Q (Merck KGaA, Darmstadt, Germany) water (5 g) within a Teflon beaker followed by the addition of 47% HF (120 mg) at room temperature, ultrasonication for 2 min to provide dissolution, and dilution with Milli-Q water (90 g). The crystal sizes and morphologies of the zeolite catalysts were observed by means of field emission scanning electron microscopy (FE-SEM; JSM-7001F, JEOL Ltd., Akishima, Tokyo, Japan). Solid-state magic angle spinning nuclear magnetic resonance (MAS NMR) data were collected using a spectrometer (AVANCEIII 600, Bruker Co., Billerica, MA, USA) operated at 600 MHz for ^1^H analyses and 119.2 MHz for ^29^Si analyses. All MAS NMR spectra were recorded at room temperature with a 4 mm diameter ZrO_2_ tube. The ^29^Si chemical shifts were determined based on that of hexamethylcyclotrisiloxane at −9.66 ppm. Dipolar-decoupling (DD) MAS NMR data were acquired using 1024 pulses with a recycle time of 30 s at a spinning rate of 10 kHz. The coke contents of the spent catalysts were determined by thermogravimetric/differential thermal analysis (TG-DTA) on a Thermo plus EVO II TG8120 (Rigaku). The temperature was raised from room temperature to 800 °C with the rate of 10 °C·min^−1^ under air flow (30 cm^3^·min^−1^). The weight loss observed from 300 to 700 °C was ascribed to coke.

### 2.2. Typical YNU-5 Synthesis Procedure

The YNU-5 zeolite was typically synthesized as follows [19,21]. Initially, an aqueous Me_2_Pr_2_N^+^OH^−^ solution (2.847 mmol·g^−1^, 11.94 g, 34.0 mmol), aqueous NaOH solution (9.048 mmol·g^−1^, 3.32 g, 30.0 mmol), aqueous KOH solution (6.075 mmol·g^−1^, 4.94 g, 30.0 mmol), colloidal silica (21.59 g; Ludox AS-40, DuPont de Nemours Inc., Wilmington, DE, USA, 40.2 wt% SiO_2_, 8.68 g SiO_2_, 144.4 mmol SiO_2_), and Milli-Q water (1.50 g) were combined in a 150 mL Teflon vessel. The vessel was tightly capped and the mixture stirred for 3 h on a hot plate while maintaining a temperature of approximately 60 °C. This procedure was essential to obtaining a clear solution. After cooling to room temperature, a **FAU**-type zeolite (Tosoh Co., Tokyo, Japan, HSZ-350HUA, 5.03 g; Si/Al = 5.5) was added and the resulting suspension was stirred for 10 min at room temperature. It should be noted that the synthesis results were found to be sensitive to the **FAU**-type zeolite manufacturer’s lot that was employed, and so the starting gel composition had to be slightly tuned depending on the lot number. For a typical example in this work, the molar composition of the starting gel was 0.265SiO_2_ (from **FAU**) – 0.735SiO_2_ (from colloidal silica) – 0.025Al_2_O_3_ (from **FAU**) – 0.17Me_2_Pr_2_N^+^OH^−^ – 0.15NaOH – 0.15KOH – 7.5H_2_O. This mixture was transferred to a 125 mL Teflon-lined stainless-steel autoclave that was subsequently sealed and allowed to stand statically for 4 days in a convection oven at 160 °C. After cooling the autoclave to room temperature, the resulting solid was separated by filtration, washed several times with de-ionized water, and dried overnight. The as-synthesized YNU-5 zeolite was obtained as a white powder (6.72 g) and was calcined at 550 °C for 6 h to remove occluded organics to give the final product (6.31 g) as a white powder (Si/Al = 9.1). In order to examine the effect of H_2_O/SiO_2_ ratio (*w*) in the starting mixture on the YNU-5 synthesis, the amount of input water was varied as follows. Input amounts were 10.50, 8.70, 6.91, 5.11, 3.31, 1.50, −0.30, and −3.91 g, where *w* values were 10.0, 9.5, 9.0, 8.5, 8.0, 7.5, 7.0, and 6.0, respectively. The negative values mean that the evaporation of excess water by stirring the mixture at approximately 60 °C on a hot plate [19,21]. 

The four representative samples used in the present study were prepared by varying the rotation rate (*x*, in rpm) of autoclaves in the oven and crystallization period (*y*, in days). The “rotation” means rotating the autoclave to mix the contents well; this technique is often used for screening experiments because it is more convenient than the typical stirring system. The rotation rate is one of the important variables for zeolite synthesis [22,23]. The YNU-5 samples were synthesized under conditions for which the (*x*, *y*) values were (0, 4), (0, 7), (20, 4), and (20, 7), and are designated herein as YFI-A, YFI-B, YFI-C, and YFI-D, respectively. The typical procedure described above corresponds to the synthesis of YFI-A.

### 2.3. Preparation of MFI-Type Zeolite Nanocrystals

The typical procedure used to prepare nanocrystals of **MFI** was as follows. An aqueous solution of Me_2_Pr_2_N^+^OH^−^ (Sachem Inc., Austin, TX, USA, 2.691 mmol·g^−1^, 1.26 g, 3.4 mmol), NaOH (11.980 mmol·g^−1^, 0.26 g, 3.0 mmol) and KOH (7.914 mmol·g^−1^, 0.38 g, 3.0 mmol), and colloidal silica (2.65 g; Ludox AS-40, DuPont de Nemours Inc., 40.2 wt% SiO_2_, 1.07 g SiO_2_, 17.8 mmol SiO_2_) was transferred into a 23 mL Teflon cup, after which the mixture was stirred at 60 °C. During this process, 0.295 g of water evaporated from the mixture, after which dealuminated **FAU**-type zeolite (0.60 g; SiO_2_: 69.7 wt%, Al_2_O_3_: 6.8 wt%, H_2_O: 23.5 wt%, Si/Al = 8.7) was added to the clear solution and the resulting suspension stirred for 10 min. The final gel composition was 1.0SiO_2_ – 0.016Al_2_O_3_ – 0.136Me_2_Pr_2_NOH – 0.12NaOH – 0.12KOH – 5.6H_2_O. This mixture was added to a 23 mL Teflon-lined stainless steel autoclave that was maintained at 160 °C in a convection oven without rotation for 7 days. The precipitated solid was separated by centrifugation, washed several times with de-ionized water, and dried overnight at 80 °C. The as-synthesized **MFI** nanocrystals (0.46 g) were calcined at 550 °C for 6 h to remove occluded organics, giving a product (0.42 g, Si/Al = 20) designated herein as MFI_nano_(20). For comparison purposes, micron-sized ZSM-5 crystals (Si/Al = 19.7), denoted as MFI_micron_(20), were obtained using a synthesis procedure previously reported in the literature [24].

### 2.4. Post-Synthesis Modification

The calcined YNU-5 samples were converted to protonated dealuminated analogues using various acid treatments. Direct dealumination of the calcined YNU-5 (typically 1.0 g) was accomplished by refluxing with 0.1–13.4 mol·L^−1^ HNO_3_ solutions (60 mL (g-sample)^−1^) in a 200 mL round bottom flask at 130 °C in an oil bath for 24 h. These conditions also stabilized the framework of the material due to Si migration [19,21]. The dealuminated versions of YFI-A, B, C, and D are referred to herein as deAl-YFI-A, B, C, and D(*n*), respectively, where *n* indicates the Si/Al ratio. In this work, as an example, deAl-YFI-A(51), deAl-YFI-B(57), deAl-YFI-C(55), and deAl-YFI-D(63) were prepared by treatment with a 2.0 mol·L^−1^ HNO_3_ solution, while deAl-YFI-C(287) was prepared with a 13.4 mol·L^−1^ HNO_3_ solution under reflux conditions.

In the case of physical mixtures of calcined YNU-5 and small amounts of MFI_nano_(20) or MFI_micron_(20), the calcined materials were first mixed after which the mixture was treated with a HNO_3_ solution as described above. These mixtures are designated as deAl-[YFI-C + 3 wt% MFI_nano_(20)] and deAl-[YFI-C + 3 wt% MFI_micron_(20)], respectively.

### 2.5. Procedure for the DTO Reaction

Each catalyst was pelletized without a binder, roughly crushed, and then sieved to obtain 500–600 μm particles. These catalyst particles (typically 100 mg) were placed in a fixed-bed reactor (a down-flow quartz tube microreactor with a 9 mm internal diameter) situated in an electric furnace. Each sample was first pretreated at 550 °C for 1 h under an air flow at 40 cm^3^(NTP) min^−1^ and then maintained at 400 °C under a He flow at 40 cm^3^(NTP) min^−1^, acting as a carrier gas. While maintaining the specimen at 400 °C, DME (at a partial pressure of 5.0 kPa) was introduced through the top of the reactor. The contact time, *W*/*F* (where *W* value is catalysts weight and *F* value is flow rate of DME), was 20 g-cat h mol^−1^ in these trials but could be varied by changing the flow rate or catalyst amount if necessary. The reactants and products were analyzed by gas chromatography (GC 2014, Shimadzu) using a DB-5 capillary column (Agilent Technologies Inc., Santa Clara, CA, USA; id 0.53 mm, length 30 m, 5.00 μm thick stationary phrase) and an HP-PLOT/Q capillary column (Agilent Technologies; id 0.53 mm, length 30 m, 40.0 μm thick stationary phase) together with a flame ionization detector.

## 3. Results and Discussion

### 3.1. Synthetic Investigations of YNU-5

In our previous paper, we reported a drastic effect of the H_2_O/SiO_2_ molar ratio (*w*) in the starting gel on the resulting zeolitic phases. Specifically, relatively water-rich conditions promoted crystallization of a zeolitic phase with a higher framework density (FD_Si_ [19]), and it was speculated that a more concentrated gel solution enhanced the degree of interaction between the OSDA molecules and the silicate. Further and more detailed investigations of the effects of the H_2_O/SiO_2_ molar ratio were performed in the present study.

**Figure 1:**
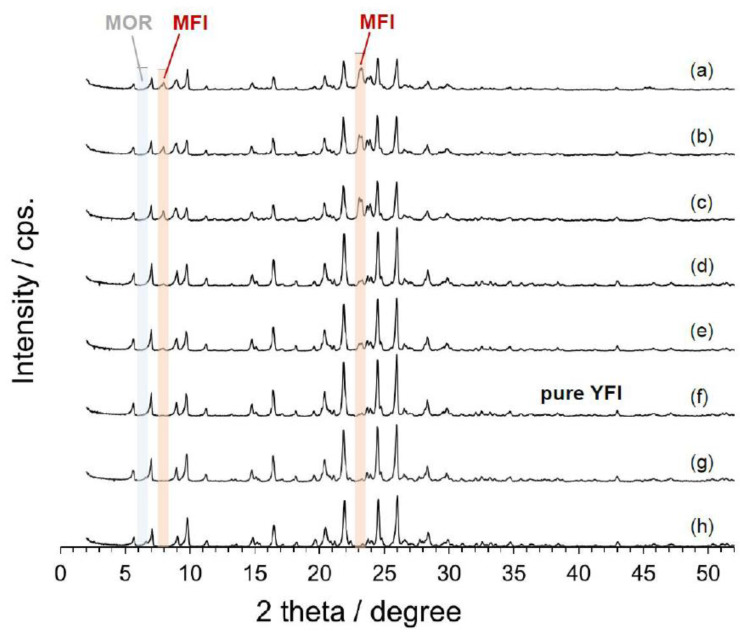
Powder X-ray diffraction patterns showing the effect of water content (*w* = H_2_O/SiO_2_ molar ratio) in the starting gel on the phase-purity of the product of YNU-5 synthesis. The *w* values in this series were (a) 10, (b) 9.5, (c) 9, (d) 8.5, (e) 8, (f) 7.5, (g) 7, and (h) 6. The starting gel with molar composition 0.265SiO_2_ (from **FAU**) – 0.735SiO_2_ (from colloidal silica) – 0.025Al_2_O_3_ (from **FAU**) – 0.17Me_2_Pr_2_N^+^OH^−^ – 0.15NaOH – 0.15KOH – *w*H_2_O was heated under static conditions at 160 °C for 4 days. The **FAU** was HSZ-350HUA (Lot #35UA8301B).

Initially, *w* was varied from 10 to 6 in 0.5 intervals, and the results are shown in Figure 1. Over this entire range, the major phase was always **YFI**. However, below a value of 10, an **MFI** phase was clearly present as a contaminant. The level of this impurity decreased with decreases in the water content, such that pure YNU-5 was obtained at *w* = 7.5. As shown in Figure 1, there were no impurity phases other than **MFI** in the range of 10–7.5. When *w* was 7.0 or smaller, a **MOR** phase began to appear although the **YFI** phase was still almost pure at *w* = 7.0. This result is consistent with the hypothesis [21] that more water-rich conditions favor the formation of materials with higher FD_Si_ values. In fact, the FD_Si_ values of **MOR** and **MFI** are 17.0 and 18.4, respectively [19].

**Figure 2:**
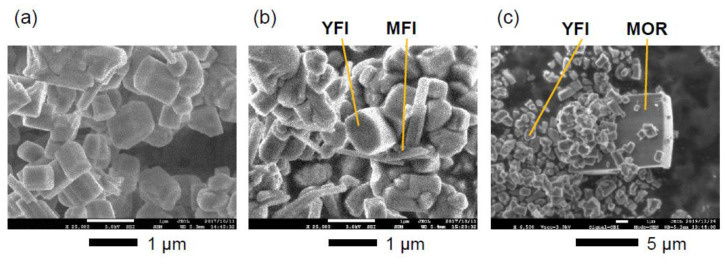
Field emission scanning electron microscopy (FESEM) images of the (**a**) pure **YFI**, (**b**) **YFI** with trace **MFI**, and (**c**) **YFI** with trace **MOR**.

The crystals of the contaminant phases were readily distinguished from the major **YFI** phase in the FE-SEM images (Figure 2). The **MFI** phase consisted of sub-micron or nano-sized pillar-like crystals (Figure 2b), whereas the **MOR** impurity comprised micron-sized crystals (Figure 2c). The fact that a slight change in the water content in the starting gel promoted the formation of impurities suggests that the structure-directing ability of the OSDA, Me_2_Pr_2_N^+^, was not very high.

**Figure 3:**
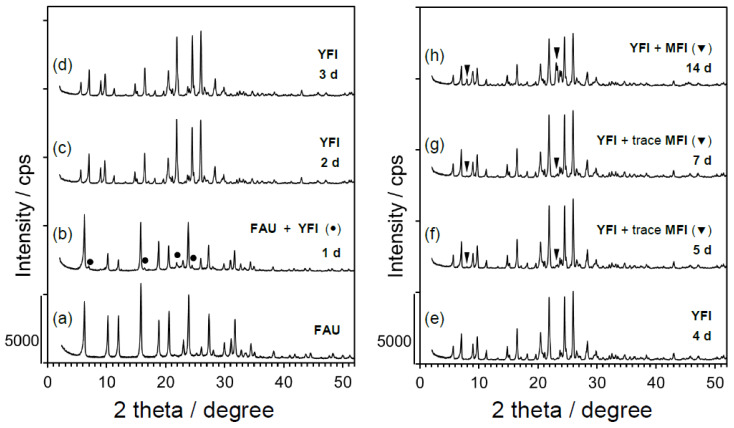
Phase changes over time as reflected in the powder X-ray diffraction patterns of (a) the starting material (**FAU**) and (b–h) as-synthesized samples crystallized under static conditions at 160 °C. The starting molar composition was 0.265SiO_2_ (from **FAU**) – 0.735SiO_2_ (from colloidal silica) – 0.025Al_2_O_3_ (from **FAU**) – 0.17Me_2_Pr_2_N^+^OH^−^ – 0.15NaOH – 0.15KOH – 7.0H_2_O. The **FAU** was HSZ-350HUA (Lot #35UA3Y02).

The effect of the crystallization period on the YNU-5 product was also investigated, and the time-course of crystallization is presented in Figure 3. The composition of starting mixture and the synthesis conditions are shown in the caption. Figure 3, line a shows the powder XRD pattern of the **FAU**-type zeolite used as the starting material, while Figure 3, line b demonstrates that the **YFI** phase began to appear while the major phase was still **FAU** after 1 day. The **FAU** phase completely disappeared and a pure **YFI** phase was observed after 2 days. After 4 days, peaks assignable to an **MFI** phase appeared and the intensity of these peaks gradually increased upon prolonged heating. In the early stage of the synthesis, it appears that the **FAU** transformed to **YFI**; however, it is more likely that the **FAU** initially dissolved and that some fragments were responsible for the crystallization of **YFI**, based on other examples of hydrothermal conversion such as from **FAU** to **MSE** [25], *BEA to **AFI** [26,27], and *BEA to *STO [28]. During the investigation on the time-course of crystallization in this work (see Figure 3), the same high level of solids recovery was consistently obtained after 2 days. Even after 1 day, a significant decrease in solid recovery was not observed, indicating that the crystallization rate of the **YFI** was much greater than the dissolution rate of **FAU**. The co-crystallized **MFI** that appeared after 4 days may not have been transformed from **YFI** but rather was generated as a result of independent nucleation from the mother gel. The FE-SEM image in Figure 2b is consistent with this hypothesis because it shows that crystals of **YFI** and **MFI** existed independently. Crystallization for a span of 2–3 days appears to have given the highest degree of phase purity. However, in the present work, we tuned the synthesis conditions so as to require crystallization times in the range of 4–7 days, and found interesting results with regard to the catalytic performance of the materials.

**Figure 4:**
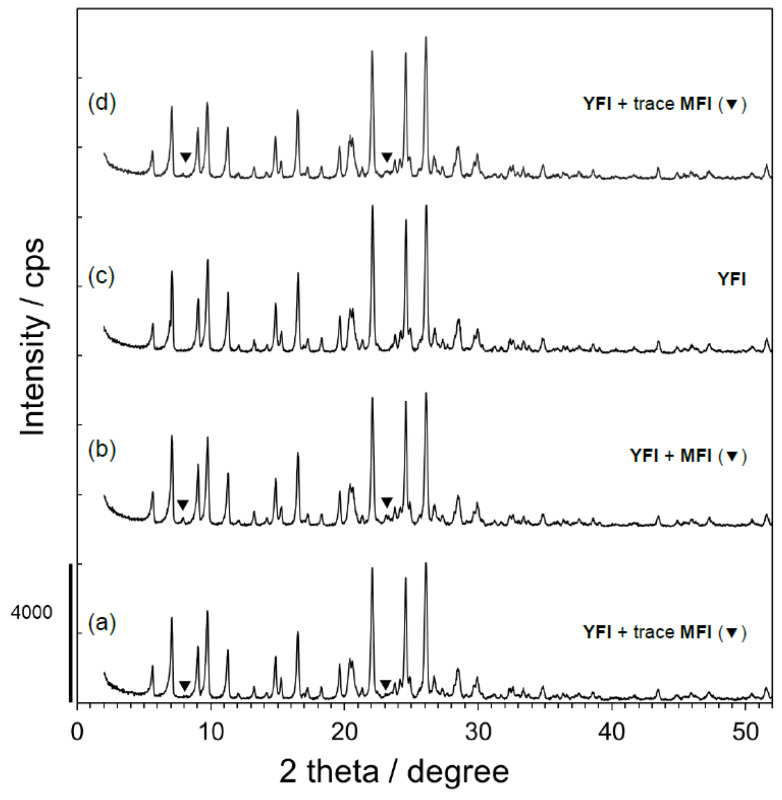
Powder X-ray diffraction patterns for calcined forms of (a) YFI-A, (b) YFI-B, (c) YFI-C, and (d) YFI-D crystallized while varying the rotation rate (*x*, rpm) and crystallization period (*y*, days), with (*x*, *y*) values of (a) (0, 4), (b) (0, 7), (c) (20, 4), and (d) (20, 7). The nomenclature for these samples is explained in Section 2.2. The starting molar composition was 0.265SiO_2_ (from **FAU**) – 0.735SiO_2_ (from colloidal silica) – 0.025Al_2_O_3_ (from **FAU**) – 0.17Me_2_Pr_2_N^+^OH^−^ – 0.15NaOH – 0.15KOH – 7.0H_2_O. The **FAU** was HSZ-350HUA (Lot #35UA3Y02).

Rotation of the synthesis vessel during the synthesis (see Section 2.2) is sometimes a key factor affecting the final products. As an example, topological changes from **MFI** to **TON**-type zeolites when using *n*-butylamine and 1,6-diaminohexane as the OSDAs were observed upon going from static to rotating conditions in a prior work [22]. Thus, to further examine the relationship between synthesis conditions and the formation of second phases, the effects of rotation and the crystallization period were also investigated in the present research. In these trials, the rotation rate (*x*, in rpm) and crystallization period (*y*, in days) were varied such that the (*x*, *y*) pairs were (0, 4), (0, 7), (20, 4), and (20, 7), while keeping the other parameters constant. The resulting samples are denoted herein as YFI-A, YFI-B, YFI-C, and YFI-D, respectively, and the results are summarized in Figure 4. These data demonstrate that the appearance of trace amounts of **MFI** as an impurity was more effectively suppressed when *x* was larger and *y* was smaller. The best results were obtained at (*x*, *y*) = (20, 4), such that the corresponding YFI-C sample comprised a pure **YFI** phase (Figure 4, line c). In addition, regardless of the rotation rate, longer crystallization times increased the probability of forming the **MFI** impurity, although the amounts of this phase remained almost negligible.

### 3.2. Effects of a Trace of MFI on the Physicochemical Properties of YFI Samples

The effects of trace amounts of **MFI**, the most frequent contaminant, on some physicochemical properties of bulk YNU-5 samples were investigated, employing the YFI-A–D that are discussed in the previous section and shown in Figure 4. Since the original material was composed of a very pure **YFI** phase, it was possible to establish the effects of contaminants by comparing this specimen with three other samples: YFI-A, B, and D. The trace amount of **MFI** did not affect the bulk chemical composition of the material as revealed by ICP analysis (Table S1).

Figure S1 presents the ^27^Al MAS NMR spectra of the YNU-5 samples (YFI-A–YFI-D) acquired immediately after removing the OSDA by calcination at 550 °C for 6 h. All these spectra are similar and the major peaks in the range of 50–60 ppm are assignable to tetrahedral aluminum, indicating that the majority of the aluminum atoms were incorporated into the framework. The minor peak at approximately 0 ppm is ascribed to octahedral Al, which tends to appear in calcined samples and was not observed in the as-synthesized YNU-5 samples. Figure S2 provides the ^29^Si MAS NMR spectra of the calcined YNU-5 samples. The spectra for samples A, B, and D (Figure S2, lines a,b,d, respectively) are seen to be similar to that produced by the pure calcined YNU-5 sample YFI-C (Figure S2, line c). It is interesting to note that the framework Si/Al ratios estimated from the ^29^Si MAS NMR spectra are slightly higher than the bulk Si/Al ratios determined by ICP analysis (Table S1), which is consistent with the ^27^Al MAS NMR results. At this stage, no obvious differences were observed among samples A–D.

**Figure 5:**
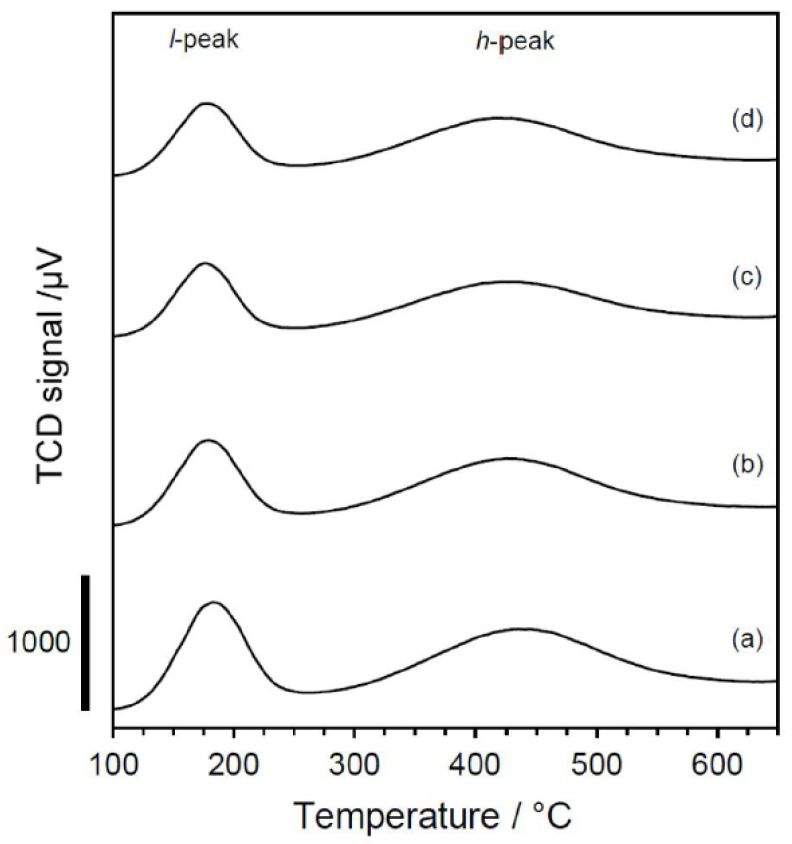
The NH_3_-temperature programmed desorption profiles for the (a) deAl-YFI-A(51), (b) deAl-YFI-B(57), (c) deAl-YFI-C(55), and (d) deAl-YFI-D(63). The acid concentrations determined from the *h* peaks are (a) 0.258, (b) 0.290, (c) 0.224, and 0.249 mmol·g^−1^, respectively. The Al levels determined by ICP analysis are (a) 0.325, (b) 0.319, (c) 0.267, and 0.301 mmol·g^−1^, respectively. As explained in Section 2.4, the dealuminated versions of YFI-A, B, C, and D are referred to herein as deAl-YFI-A, B, C, and D(*n*), respectively, where *n* indicates the Si/Al ratio.

To assess the application of these materials as solid acid catalysts, samples A–D (in calcined form) were dealuminated with 2 mol·L^−1^ HNO_3_ in a 130 °C oil bath for 24 h [20]. It was confirmed by XRD that the samples maintained a high degree of crystallinity after dealumination (see Figure S3). Figure 5 shows the ammonia temperature-programmed desorption (NH_3_-TPD) profiles for these same samples along with the acid amounts (that is, the number of acid sites) estimated from the so-called *h*-peak for each material [29,30]. The discrepancy between the Al content (0.224 mmol-Al g^−1^) determined by ICP analysis (see Figure 5 caption) and the number of acid sites (0.249 mmol·g^−1^) estimated by NH_3_-TPD for the pure **YFI** (YFI-C) was not unexpected and was in good agreement with the results of our previous studies concerning other zeolites [31]. Underestimation of acid amounts by NH_3_-TPD compared to the Al content determined by ICP analysis is very common and there are some possible reasons for this. These include the existence of non-acidic extra-framework Al atoms [32], inaccessible tetrahedral Al sites in the framework, and residual K^+^ at [Si–O–Al]– sites in the framework. These effects are typical in the case of the parent samples before dealumination; however, the number of acid sites in each dealuminated **YFI** sample was closer to the bulk Al content, together with a K/Al molar ratio of less than 0.01. At the dealuminated stage, the NH_3_-TPD profiles are very similar between the YFI-A–D specimens, regardless of the effects discussed immediately above.

Overall, the similarities in the Si/Al ratios, framework characteristics, and even acid properties indicate that the presence of trace amounts of impurities had no obvious effects on the physicochemical properties of the YNU-5 in the case that the extent of contamination was as low as in these specimens.

### 3.3. DTO Reaction over the YFI Catalyst

#### 3.3.1. Effect of MFI as a Trace Contaminant

The conversion of DME is an attractive alternative approach to the sustainable production of olefins and could replace the classical routes based on the thermal cracking of ethane [33]. DME is commonly obtained from the dehydration of methanol acquired from non-fossil resources such as natural gas, coal, biomass, or waste gasification/reforming [34]. Due to its low cost and high hydrocarbon selectivity, the DTO reaction has become increasingly attractive in recent years [35,36]. The present work focused on performing this reaction catalyzed by zeolite-based solid acid materials with the expectation of obtaining high selectivity for propylene and butenes [31]. In the previous section, we reported almost no effect of trace contamination on the physicochemical properties of these catalysts. In contrast, small amounts of impurities had profound effects on the DTO reaction when using deAl-YFI-C(55) (that is, a pure **YFI** phase) and deAl-YFI-B(57) (**YFI** containing approximately 3 wt% of an **MFI** phase).

**Figure 6:**
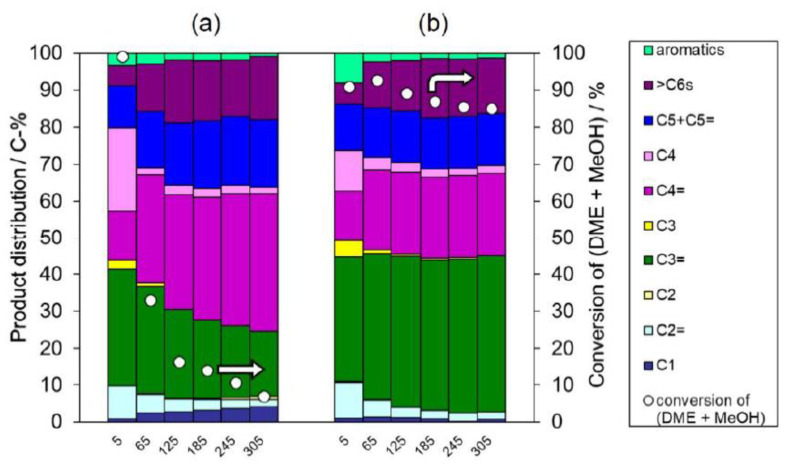
Results from the dimethyl ether (DME)-to-olefin (DTO) reaction over the (**a**) deAl-YFI-C(55) and (**b**) deAl-YFI-B(57). Pretreatment conditions: 550 °C, 1 h under an air flow of 40 cm^3^(NTP) min^−1^. Reaction conditions: catalyst weight, 100 mg; *W*/*F* = 20 g-cat h mol^−1^; pellet size, 500–600 μm; He flow rate, 40 cm^3^(NTP)·min^−1^; temperature, 400 °C. As explained in Section 2.4, the dealuminated versions of YFI-A, B, C, and D are referred to herein as deAl-YFI-A, B, C, and D(*n*), respectively, where *n* indicates the Si/Al ratio. Coke amounts in the spent catalysts estimated by thermogravimetric analyses were (**a**) 91 and (**b**) 105 mg (g-cat)^−1^.

Figure 6a,b shows the conversions as well as product distributions over deAl-YFI-C(55) and deAl-YFI-B(57), respectively. The conversions were high after a short TOS (5 min) over both catalysts. However, in the case of the deAl-YFI-C(55), a rapid decrease in conversion was observed as the TOS increased. In fact, conversion decreased to <40% at a TOS of 65 min. This result demonstrates that the pure YNU-5 catalyst deactivated rapidly during the DTO reaction. After the removal of acid sites on the external surfaces and pore mouths (preferentially the 12-ring pores) by the acid treatment, the remaining acid sites on the pore mouths of isolated 8-rings may have directly promoted coke formation [21]. The detailed location of the remaining acid site after deep dealumination will be investigated in future work. The most surprising finding was that this phenomenon changed when deAl-YFI-B(57) was used as the catalyst, such that the deactivation of the YNU-5 was greatly suppressed and the conversion remained high even at a TOS of 305 min. The thermogravimetric analysis of the spent catalysts indicated there was no significant difference in the amount of coke deposition between deAl-YFI-C(55) and deAl-YFI-B(57) in Figure 6. This suggests that the long-lived active sites in deAl-YFI-B(57) are not influenced by currently observed coke formation even though some different active sites are deactivated.

**Figure 7:**
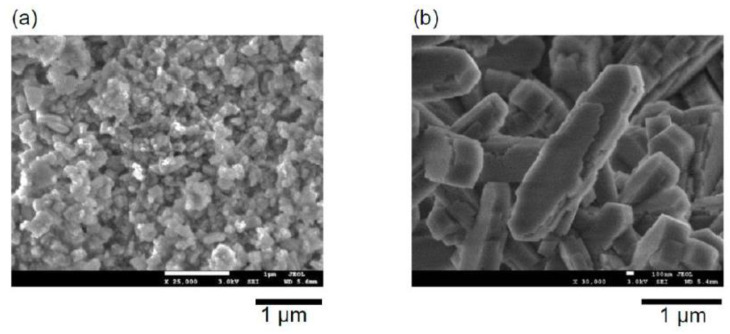
Field emission scanning electron microscopy images of the (**a**) MFI_nano_(20) and (**b**) MFI_micron_(20). The nomenclature for these samples is explained in Section 2.3.

To determine if the small amounts of impurities were responsible for the significant changes in performance, we intentionally mixed YFI-C (that is, pure YNU-5) with trace amounts of MFI_nano_(20) or MFI_micron_(20), dealuminated using a standard technique to compare with deAl-YFI-B(57), and used the resulting deAl-[YFI-C + 3 wt% MFI_nano_(20)] and deAl-[YFI-C + 3 wt% MFI_micron_(20)] (as defined in Section 2.4) as catalysts for the DTO reaction. Note that the abbreviations MFI_nano_(20) and MFI_micron_(20) used here are as defined in Section 2.3. The particle sizes and morphologies of these samples were confirmed by FE-SEM, as shown in Figure 7. The MFI_nano_(20) or MFI_micron_(20) was added at 3 wt% based on physically mixing pure calcined YNU-5 (YFI-C) with MFI_micron_(20) so that the YFI-C:MFI_micron_(20) weight ratio was 97:3, 95:5, or 90:10. Comparing the YFI-B to these mixtures, the amount of the **MFI** phase in the YFI-B was estimated to be 3 wt%.

**Figure 8:**
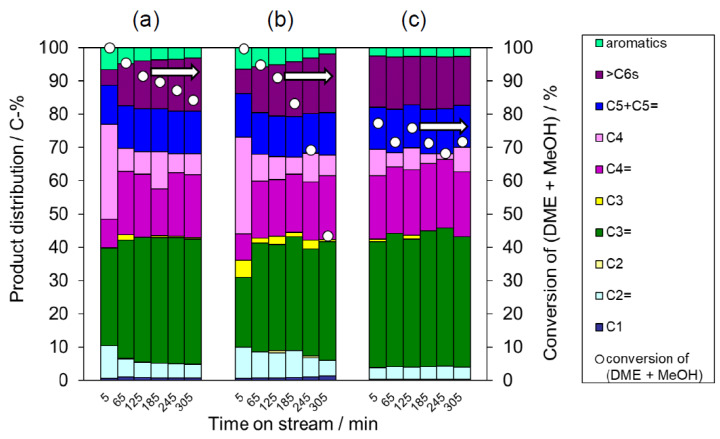
Results from the DTO reaction over the (**a**) deAl-[YFI-C + 3 wt% MFI_nano_(20)] (Si/Al = 62), (**b**) deAl-[YFI-C + 3 wt% MFI_micron_(20)] (Si/Al = 62), and (**c**) deAl-[Si_3_N_4_ + 3 wt% MFI_nano_(20)]. The nomenclature for these samples is explained in Section 2.4 for (**a**) and (**b**), and Section 3.3.1 for (**c**). Pretreatment conditions: 550 °C, 1 h under air at a flow rate of 40 cm^3^(NTP) min^−1^. Reaction conditions: catalyst weight, 100 mg; *W*/*F* = 20 g-cat h mol^−1^; pellet size, 500–600 μm; He flow rate, 40 cm^3^(NTP)·min^−1^; temperature, 400 °C. Coke amounts in the spent catalysts estimated by thermogravimetric analyses were (a) 114 and (b) 94 mg (g-cat)^−1^.

To allow a comparison with the deAl-YFI-B(57) (Figure 6b), the catalytic performances of the physical mixtures are summarized in Figure 8a,b. During the initial stage of the reaction (TOS < 5 min), the **YFI** phase evidently acted as the main catalyst. However, the deAl-YFI-C(55) was rapidly deactivated due to coke formation (Figure 6a). After this initial stage (TOS > 5 min), it appears that the **MFI** phase primarily catalyzed the reaction. Compared to the data for deAl-YFI-C(55) in Figure 8a, it is evident that deactivation was effectively suppressed when using the deAl-[YFI-C + 3 wt% MFI_nano_(20)] (Si/Al = 57, Figure 8a) or deAl-[YFI-C + 3 wt% MFI_micron_(20)] (Si/Al = 62, Figure 8b). Only minimal deactivation was observed in both cases at TOS values between 5 and 185 min. Unexpectedly, with further increases in TOS, a significant decrease in conversion occurred when using the deAl-[YFI-C + 3 wt% MFI_micron_(20)]. In contrast, the conversion was maintained at 80% at a TOS of 305 in the case of the nanosized **MFI** deAl-[YFI-C + 3 wt% MFI_nano_(20)]. In this case, the selectivity for propene (a C3=) greatly increased as well. It should be noted that the samples made via the intentional physical mixing of MFI_nano_ showed very similar catalytic performance (Figure 8a) to that in Figure 6b, which suggests that trace levels of MFI_nano_ were responsible for the long life of the deAl-YFI-B(57). The fact that typical micron-sized MFI particles exhibited a lesser effect (Figure 8b) indicates the advantage of the nano-sized MFI contaminant. A control experiment was carried out as follows. MFI_nano_(20) was physically mixed with inert material Si_3_N_4_ at the weight ratio of 3:97 after which the mixture was treated with a HNO_3_ solution as described in Section 2.4. The solid mixture was designated as deAl-[Si_3_N_4_ + 3 wt% MFI_nano_(20)] and was used as catalyst for DTO reaction (Figure 8c). As a result, the deAl-[Si_3_N_4_ + 3 wt% MFI_nano_(20)] showed stable activity as well as the similar product distribution pattern to that in Figure 8a, which further confirms this extraordinary advantage of MFI_nano_(20). The most interesting aspect of these data is that the formation of a trace amount of co-crystallized MFI along with the crystallization of YNU-5 produced similar results to the material with added MFI_nano_.

#### 3.3.2. Catalytic Characteristics of Pure YNU-5

**Figure 9:**
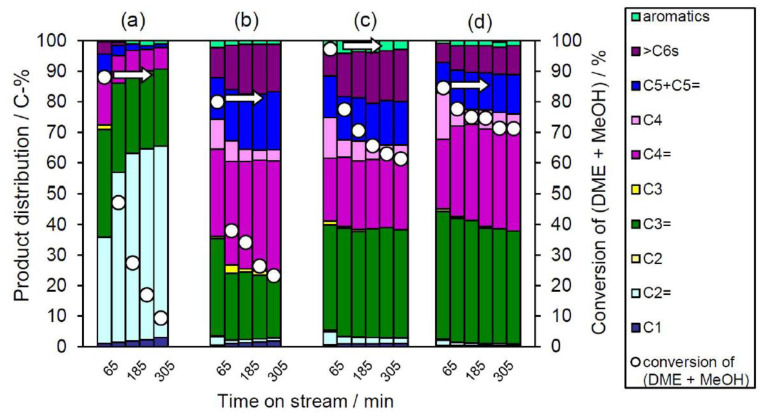
Results from the DTO reaction over the (**a**) SSZ-13 (Si/Al = 136), (**b**,**c**) deAl-YFI-C(287), and (**d**) beta (Si/Al =250). Pretreatment conditions: 550 °C; 1 h under an air flow of 40 cm^3^(NTP)·min^−1^. Reaction conditions: catalyst weight, 100 mg; *W*/*F* = 20 g-cat h mol^−1^ with the exception of (**c**) where *W*/*F* = 79 g-cat h mol^−1^; pellet size, 500–600 μm; He flow rate, 40 cm^3^(NTP) min^−1^; reaction temperature, 400 °C. The nomenclature for deAl-YFI-C(287) is explained in Section 2.4. Coke amounts in the spent catalysts estimated by thermogravimetric analyses were (**a**) 91, (**b**) 29, (**c**) 25, and (**d**) 19 mg (g-cat)^−1^.

Because we succeeded in obtaining very pure YNU-5, we investigated the intrinsic catalytic features of this 12-12-8-ring zeolite by comparing its catalytic performance with those of 8-ring and 12-ring zeolites. SSZ-13 (having a **CHA** topology with an 8-8-8-ring pore system) and beta (having a *BEA topology with a 12-12-12-ring pore system) were used as model catalysts for the DTO reaction. Figure 9 summarizes the product distributions obtained from this reaction when catalyzed by the SSZ-13 (Si/Al = 136, Figure 9a), deAl-YFI-C(287) (Figure 9b,c), and beta (Figure 9d) materials. It is obvious that the SSZ-13 gave very high C2= selectivity as high as 35% at a conversion of 88% (Figure 9a). In contrast, the C3= and C4= selectivities obtained from the beta were remarkably high, with a combined [C3= + C4=] selectivity of approximately 65% in conjunction with a conversion of 85% (Figure 9d). Thus, the product distributions tended to reflect the pore system in the catalyst. To obtain comparable conversion levels, the *W*/*F* value was increased by a factor of 4 when using the YNU-5, with the results presented in Figure 9c. The DTO reaction catalyzed by the highly dealuminated YNU-5 (deAl-YFI-C(287)) gave a product distribution pattern that was very similar to that obtained from the reaction over the beta specimen (Si/Al = 250) (Figure 9c,d). At a conversion of 80%, the selectivity for [C3= + C4=], which are the products in growing demand at present [37,38,39], was close to 60% (Figure 9c), suggesting that this material could have practical applications. Thus, the behavior of the highly dealuminated YNU-5 was closer to that of a 12-ring system rather than an 8-ring zeolite catalyst. This result could assist in determining the locations of acid sites in the YNU-5 catalyst, and more detailed investigations of these effects are currently underway in our laboratory.

## 4. Conclusions

Following the successful synthesis and structural determination of YNU-5 having a **YFI** framework [19], it became important to assess several aspects of this material with regard to potential catalytic applications. This included defining the factors that affect crystallization [21], preparing a high-silica YNU-5 catalyst with a stable framework [21], and establishing the relationship between very small amounts of impurity phases and catalytic performance. The present study examined the first and third issues. During the synthesis investigation, it was found that a very slight amount of an impurity phase tended to be formed along with the desired product YNU-5. This minor phase was very often **MFI**. The phase selection was sensitive to the levels of water in the synthesis mixture, such that a pure phase could be produced but it was also possible to intentionally form specific trace impurities. Dealuminated pure YNU-5 showed rapid deactivation at TOS values greater than 5 min, while the presence of low levels of ZSM-5 as an impurity reduced the extent of deactivation. At a TOS of 305 min, conversions up to 80% were observed. During the initial reaction stage, the YNU-5 acted as the primary catalyst and showed selectivity for propene as high as 30%. When TOS was between 5 and 305 min, ZSM-5 was the main catalyst and provided selectivity for propene up to 40%. This enhanced selectivity was observed only in the case that the ZSM-5 comprised nanoparticles. It is worth noting that the ZSM-5 impurity in the synthesis system of YNU-5 was unlike typical ZSM-5 specimens in that it appeared in the form of nano-sized particles. The presence of this nanoparticle contaminant phase evidently improved the performance of the catalytic system during the DTO reaction. Based on the product distribution obtained using highly dealuminated, very pure YNU-5 as a solid acid catalyst, this material behaves more like a 12-ring zeolite. The highly dealuminated YNU-5 also shows increased selectivity for high value C3 and C4 olefins, which suggests potential practical applications.

## Supplementary Materials

The following are available online at https://www.mdpi.com/1996-1944/13/9/2030/s1, Table S1: Elemental analysis and related data of YFI samples. Figure S1: ^27^Al magic angle spinning nuclear magnetic resonance (^27^Al MAS NMR) spectra obtained from the (a) YFI-A, (b) YFI-B, (c) YFI-C, and (d) YFI-D. Figure S2: ^29^Si MAS NMR spectra obtained from the (a) YFI-A, (b) YFI-B, (c) YFI-C, and (d) YFI-D. Figure S3: Powder X-ray diffraction patterns obtained from the (a) deAl-YFI-A(51), (b) deAl-YFI-B(57), (c) deAl-YFI-C(55), and (d) deAl-YFI-D(63).
