# Supplementary material for: The Synthesis of YNU-5 Zeolite and Its Application to the Catalysis in the Dimethyl Ether-to-Olefin Reaction
Source: Materials (Basel). 2020 Apr 26;13(9):2030. doi: 10.3390/ma13092030 (PMC7254336; doi:10.3390/ma13092030)
Supplement: Supplementary file 1 [file materials-13-02030-s001.pdf]

materials

Supplementary Information

The Synthesis of YNU-5 Zeolite and Its Application to the Catalysis in the Dimethyl Ether-to-Olefin Reaction

Table S1. Elemental analysis and related data of YFI samples.

Sample[a]	Si[b] /mmol·g^{-1}	Al[b] /mmol·g^{-1}	Na[b] /mmol·g^{-1}	K[b] /mmol·g^{-1}	Si/Al[c]	Si/Al[d]
YFI-A	14.87	1.537	0.211	0.864	9.7	13.5
YFI-B	14.01	1.468	0.160	0.761	9.5	14.1
YFI-C	14.44	1.564	0.116	0.784	9.2	15.0
YFI-D	14.43	1.442	0.176	0.799	10.0	14.1

a. The nomenclature for the samples is explained in the Section 2.2.

b. Amounts in the bulk materials were determined by means of inductively coupled plasma-atomic emission spectrometry (ICP-AES).

c. The Si/Al molar ratios in the bulk materials based on ICP analyses.

d. The Si/Al molar ratios in the framework based on 29Si magic angle spinning nuclear magnetic resonance (29Si MAS NMR) spectra as shown in Figure S2.

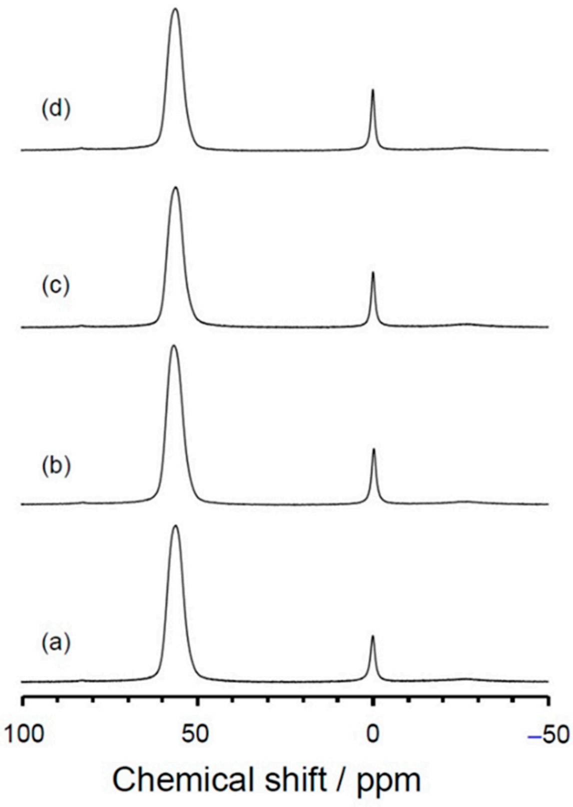

Figure S1. ^{27}Al magic angle spinning nuclear magnetic resonance (^{27}Al MAS NMR) spectra obtained from the calcined (a) YFI-A, (b) YFI-B, (c) YFI-C, and (d) YFI-D.

The nomenclature for these samples is explained in the Section 2.2.

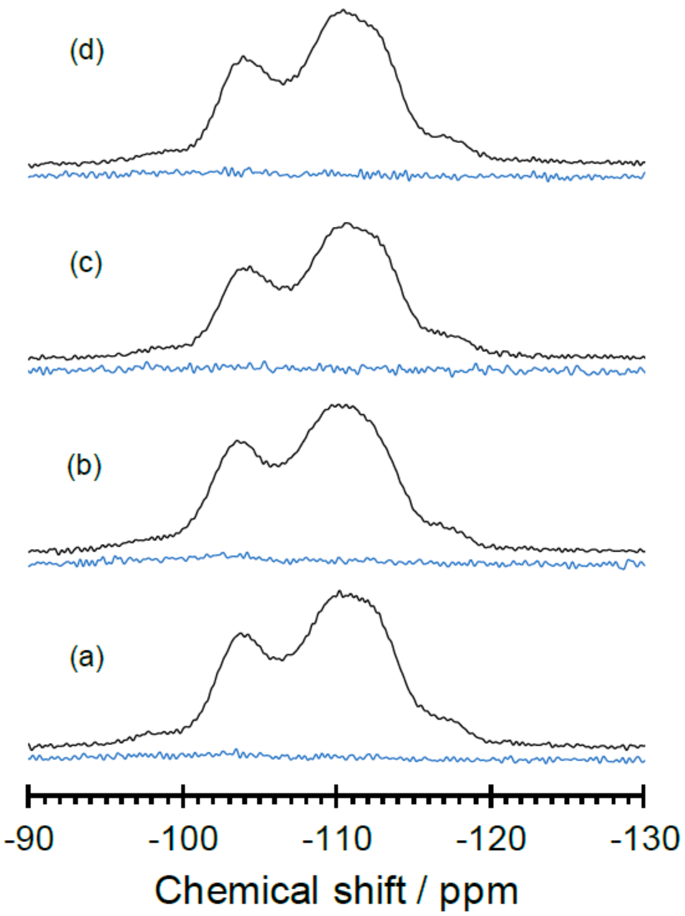

Figure S2. ^{29}Si MAS NMR spectra obtained from the (a) YFI-A, (b) YFI-B, (c) YFI-C, and (d) YFI-D.

The blue lines are ^{29}Si CPMAS NMR spectra. The nomenclature for these samples is explained in the Section 2.2.

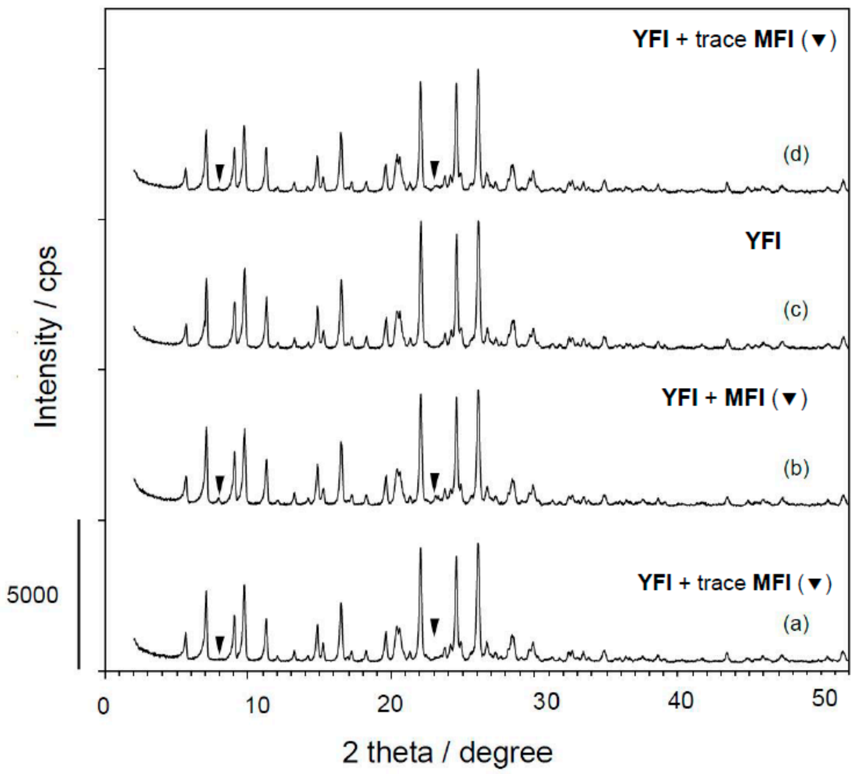

Figure S3. Powder X-ray diffraction patterns obtained from the (a) deAl-YFI-A(51), (b) deAl-YFI-B(57), (c) deAl-YFI-C(55), and (d) deAl-YFI-D(63).

The nomenclature for these samples is explained in the Section 2.4.
